# Fanconi Bickel syndrome: clinical phenotypes and genetics in a cohort of Sudanese children

**DOI:** 10.1186/s13633-020-00091-5

**Published:** 2020-11-23

**Authors:** Salwa A. Musa, Areej A. Ibrahim, Samar S. Hassan, Matthew B Johnson, Asmahan T. Basheer, Ali M. Arabi, Mohamed A. Abdullah

**Affiliations:** 1 Pediatric Endocrinology Unit, Gaafar Ibn Auf children Hospital, Khartoum, Sudan; 2 Institute of Biomedical and Clinical Science, College of Medicine and Health, University of Exeter, Exeter, UK; 3 Sudan Childhood Diabetes Center, Khartoum, Sudan; 4 Gastroenterology and Endocrinology Units Gaafar Ibn Auf Children Hospital & Faculty of Medicine, University of Khartoum, Khartoum, Sudan

**Keywords:** Fanconi Bickel syndrome, GLUT2, SLC2A2, children, Sudan, Sub-Saharan Africa

## Abstract

**Background:**

Fanconi-Bickel syndrome (FBS) is a rare condition of carbohydrate metabolism, caused by a recessive defect in the facilitative glucose transporter GLUT2 encoded by the *SLC2A2* gene and characterized by a wide spectrum of phenotypical features. There is a paucity of reported data on FBS from Sub-Saharan Africa. Here, we describe the clinical, biochemical and genetic characteristics of our patients with FBS from Sudan, a country with a high consanguinity rate.

**Patients & methods:**

Eleven patients from ten unrelated Sudanese families were included. Clinical & biochemical data were documented and imaging studies done including bone survey and abdominal ultrasound. Liver biopsy was done to confirm the pathological diagnosis in 45% of cases and molecular genetics was performed through contribution with the Exeter genomics laboratory for ten patients.

**Results:**

Reported consanguinity was 70% among our patients. Growth was significantly impaired at presentation with mean weights of (-5.3 ± 1.8) SD and heights (-5.4 ± 2.5) SD. Severe chest deformity was present in (27%) and all patients showed features of rickets at presentation. Three patients had neonatal diabetes requiring insulin therapy of which one has been reported before. Six families lost undiagnosed siblings with similar clinical presentations. We identified a total of four homozygous pathogenic *SLC2A2* variants in our patients, one of whom had a novel mutation.

**Conclusions:**

FBS is not uncommon in Sudan where there is a high rate of consanguinity. Many cases are likely missed because of variable presentation and lack of public and professionals’ awareness. This is the first series to describe this condition from Sub-Saharan Africa.

## Introduction

Fanconi-Bickel Syndrome (FBS; OMIM 227,810), is a rare autosomal recessive disorder of carbohydrate metabolism which was first described by Fanconi and Bickel in 1949 when they identified a combination of tubular nephropathy and glycogen storage disease in a Swiss boy [1]. It is characterized by hepato-renal accumulation of glycogen, proximal renal tubular dysfunction, and impaired utilization of glucose and galactose due to defect in the glucose transporter GLUT2 encoded by *SLC2A2* [2]. Common features at presentation include failure to thrive, protuberant abdomen & hepatomegaly secondary to glycogen accumulation, glucose, and galactose intolerance, fasting hypoglycaemia and postprandial hyperglycemia, proximal tubular nephropathy as evidenced by glycosuria, phosphaturia, aminoaciduria and hypophosphatemia leading to subsequent features of rickets [3]. Presentation is heterogeneous making a clinical diagnosis challenging, especially where healthcare resources are scarce. As presentation is varied, a high index of suspicion is needed to diagnose these cases. It is mainly based on the clinical symptoms, radiological and biochemical features of rickets, laboratory data of renal tubular dysfunction, and resultant metabolic acidosis and accumulation of glycogen on the liver or renal biopsy. The clinical diagnosis is confirmed by the presence of a pathogenic variant in *SLC2A2*. Treatment is largely supportive and pointed towards the management of rickets, acid-base disturbances, and glucose metabolism.

Many scattered cases have been reported worldwide from different ethnic groups, but little is known about cases from Sub-Saharan Africa [4]. This is the first series to be reported from Sudan since the first case with neonatal diabetes was published [5]. In this series, we aimed to describe the clinical and biochemical features of FBS at presentation as well as genetic mutations.

## Patients and methods

This was a cross-sectional, descriptive hospital-based study. Records of all patients who were diagnosed as having FBS in the paediatric endocrinology and gastroenterology units at Gaafar Ibn Auf Children Teaching Hospital in Khartoum, the capital city, were reviewed. This is the main tertiary care centre for Sudan which gets referrals from all over the country. Data including age, sex, ethnic group, clinical presentation, laboratory, and radiological findings as well as liver biopsy findings to confirm the pathological diagnosis were obtained in addition to the management. Genetic testing was done in the University of Exeter UK Genomic laboratory for all patients except one who had a suggestive clinical and biochemical features supported by characteristic findings on liver biopsy. The study was approved by the hospital’s ethical committee and written consent was obtained from the parents.

### Genetic testing

Genetic testing was undertaken on purified whole blood-derived DNA either by Sanger sequencing of all coding and flanking intronic regions of *SLC2A2* (NM_000340), or by targeted Next-Generation Sequencing of all coding and flanking intronic regions of *SLC2A2* as part of a panel of genes causing monogenic diabetes (full information available at www.diabetesgenes.org). *SLC2A2* variants identified were assessed against the American College of Medical Genetics guidelines for variant interpretation.

## Results

The clinical and genetic findings are summarized in Table 1.

**Table 1:** Clinical, biochemical and genetic characteristics of FBS at presentation

PATIENTS	1	2	3	4	5	6	7i^a^	7ii^a^	8	9	10
Birth weight (Kg)	2.3	2.1	NA	NA	2.2	NA	3.3	2.3	NA	2	2.2
Age at first symptoms ^b^	5	1	2	2	4	2	2	2	5	7	2
Age at presentation ^b^	10	16	2^c^	4^c^	11	10	36	2	6^c^	7	9
Sex	F	F	M	M	F	M	M	F	M	M	F
Consanguinity	Yes	Yes	Yes	Yes	No	Yes	No	No	Yes	Yes	Yes
Hepatomegaly	Yes	Yes	Yes	Yes	Yes	Yes	Yes	Yes	Yes	Yes	Yes
Polyuria	Yes	Yes	Yes	Yes	Yes	No	Yes	No	Yes	Yes	Yes
Features of rickets	Yes	Yes	Yes	Yes	Yes	Yes	Yes	Yes	Yes	Yes	Yes
Weight (kg)	5.3	4	4	3	4.2	4.5	8.4	3.7	3.5	4.5	4.7
Weight (SD)	-4.0	-8.2	-2.6	-7.0	-6.4	-6.3	-4.5	-2.5	-7.1	-5.4	-4.8
Height (cm)	63	58	55.5	NA	NA	57	72	48	54	NA	60
Height (SD)	-3.0	-9.5	-1.6	NA	NA	-7.1	-6.5	-4.6	-6.4	NA	-4.2
Glycosuria	++	NA	NA	+++	+++	NA	++	++++	++	++	++
Proteinuria	NA	NA	NA	+	NA	NA	NA	NA	+	+	+
FBS (mg/dl)	43	45	NA	46	60	92	37	84	29	NA	38
2 hr PPG (mg/dl)	309	212	230	448	302	NA	205	103	213	182	273
S. Ca (mg/dl)	9.3	10.4	10.1	12.9	9	9.2	10	8.4	7.9	8.8	9.2
S.Po4 (mg/dl)	1.5	2.3	2	2.7	2.3	2.4	2.8	2.8	1.9	2.9	1.8
S. ALP (IU/L)	1297	1410	2510	1857	1380	498	752	1371	1979	NA	NA
Acid base disturbance	MA	MA	MA	MA	MA	MA	MA	MA	CMA	MA	CMA
Liver Biopsy (+ VE for Glycogen)	NA	+VE	ND	+VE	NA	+VE	+VE	+VE	ND	ND	ND
Homozygous *SLC2A2* pathogenic variant	c.157C > T, p.(Arg53Te)	ND	c.157C > T, p.(Arg53Ter)	c.157C > T, p.(Arg53Ter)	c.735C > A, p.(Tyr245Ter)	c.320G > A, p.(Gly107Asp)^d^	c.1171-2A > G, p.?	c.1171-2A > G, p.?	c.157C > T, p.(Arg53Ter)	c.157C > T, p.(Arg53Ter)	c.157C > T, p.(Arg53Ter)

*Abbreviations: NA *not available, *FBG *Fasting blood glucose, *PPG *Post prandial glucose, *MA* Metabolic acidosis, *CMA* Compensated metabolic acidosis, *ND *Not done

^a^ (7i,7ii): Two siblings, ^b^ Age in months, ^c^ Cases of neonatal diabetes, ^d^ Novel mutation

### Clinical findings

Eleven patients were enrolled in this series, from whom two were siblings. The mean age of symptom onset was 3.2 1.8 months (range 1.07.0), while the mean age at diagnosis was 10.3 9.4 months (range 2.036.0). Reported consanguinity was (70%) and all families originated from Afro-Asian tribes in Western Sudan where there is a high rate of consanguineous marriages. Abdominal distension was the main concern that brought all the children to medical attention (100%), followed by polyuria which was the earliest symptoms to be noticed by parents in 8 patients (82%). Delayed dentition and chest deformities were among the other chief complaints. Family history was significantly positive for the same features of FBS and six families had one or two siblings who died undiagnosed. Growth was significantly impaired in almost all patients with mean weights (5.31.8) SD and heights 5.42.5) SD. The birth weight was documented to be low in 54% of patients (mean 2.20.1 Kg), and considered to be low in the rest according to their parents as these patients delivered at home, however, one had a normal birth weight.

**Fig. 1:**
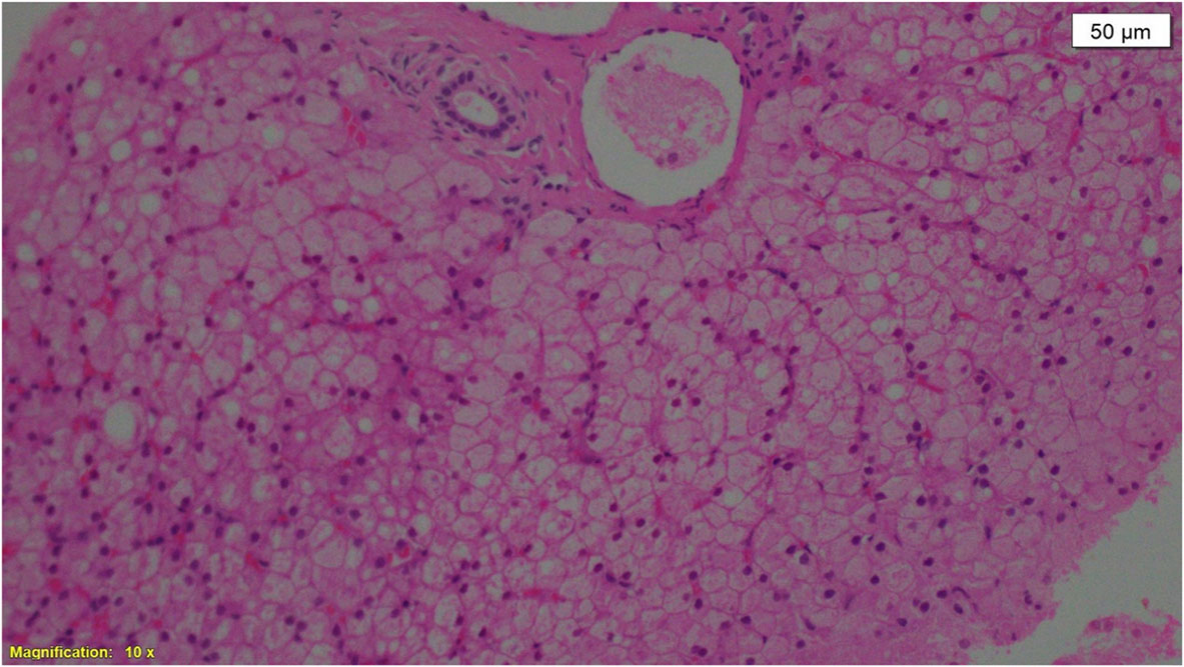
Liver biopsy showed normal portal tract, liver cells arranged in trabeculae, cytoplasm is pale and distended with PAS (Periodic Acid-Schiff) positive, diastase glycogen sensitive suggestive of glycogen storage disease

Features of rickets were prominent at presentation and severe chest deformity was present in three patients while severe abdominal distention was evident in all patients at presentation. The liver enlargement was clinically documented in all patients and later confirmed by abdominal ultrasound. Bone profile showed features of hypophosphatemic rickets with normal calcium (mean 9.6 ± 1.3), (NR = 8-10.5), low phosphate (mean 2.2 mg ± 0.46) (NR = 2.7–4.8) and high alkaline phosphatase (mean 1450.4 ± 612 U/L) (NR = 104-345). Metabolic acidosis was confirmed in all patients at presentation and eight patients (73%) showed different degrees of glycosuria and mild proteinuria. Galactose level was not checked. Six patients (54%) had fasting hypoglycemia (mean FBG 52.67 ± 21.8) mg/dl (NR = 70-99) and eight (73%) showed postprandial hyperglycemia (mean PPG 247.7 ± 92) mg/dl (NR up to 140). In three of these, the age was 6 months or less at presentation indicating neonatal diabetes. A clinical diagnosis of FBS was made for all patients and then confirmed by liver biopsy in 45% of patients showing glycogen accumulation (Fig. 1). Patients were treated with oral phosphate and vitamin D for hypophosphatemic rickets and oral bicarbonate solution for acidosis. Corn starch was added for all patients at diagnosis to prevent morning hypoglycemia while small doses of NPH insulin were given to treat diabetes.

### Genetic analysis results

We identified a total of 4 homozygous pathogenic *SLC2A2* variants in the 10 individuals where genetic testing was possible (Table 1). The p.(Arg53Ter) nonsense variant was identified in 6 individuals from 6 families (Patients 1, 3, 4, 8, 9 and 10). This variant results in a premature termination codon in exon 3/11 and therefore the likely loss of GLUT2 expression via nonsense-mediated decay. An additional nonsense variant resulting in a premature termination codon in exon 6/11, p.(Tyr245Ter), was identified in patient 5 and is also likely to result in loss of GLUT2 expression due to nonsense-mediated decay. We also identified a homozygous variant, c.1171-2A > G, affecting the canonical splice acceptor site in intron 9 and likely to resulting in aberrant splicing (siblings 7i and 7ii). A novel missense variant, p.(Gly107Asp) was identified in patient 6 and assessment against the American College of Medical Genetics guidelines resulted in the variant being classified as likely pathogenic. This was based on the variant not being present in the GnomAD database of 125,673 individuals, multiple lines of *in silico* evidence supporting pathogenicity (SIFT, PoltPhen and Align GVGD as well as having a Consurf score of 9) and the clinical diagnosis and liver biopsy of the patient being in keeping with a diagnosis of Fanconi Bickel Syndrome.

## Discussion

 FBS is a rare clinical syndrome caused by recessively inherited pathogenic variants in *SLC2A2* encoding GLUT2 (SLC2A2). The exact incidence and prevalence is not known but many pathogenic variants in *SLC2A2* from different ethnic groups have been identified, of which around half are novel. Data from Sub-Saharan Africa (apart from one case of neonatal DM, reported from Sudan), are not available [2, 4, 5].

Since the first case of FBS was reported, many additional cases have been observed with variable clinical presentations and many attempts have been made to explain the pathophysiology of this syndrome. The loss of function of GLUT2 can explain most of the characteristic clinical manifestation seen in patients with FBS. Normally, GLUT2 facilitates glucose diffusion in and out of liver cells. In patients with FBS, decreased uptake of glucose by the liver cells, leads to postprandial hyperglycemia, which is further exacerbated by low insulin secretion due to defective glucose sensing in the pancreatic ß cell. Concomitantly, defective transport of glucose out of the cells leads to increase intracellular glucose and inhibits glycogen degradation that eventually leads to hypoglycemia in the fasting state and subsequent hepatomegaly. Renal loss of glucose due to defective transport of glucose and galactose across the basolateral membrane of tubular cells contributes more to hypoglycemia and renal glycogen accumulation, further disrupting other tubular functions leading to tubular nephropathy and nephromegaly. Impairment absorption of glucose from enterocytes and carbohydrate accumulation leads to the malabsorption and gastroenteritis symptoms seen in some patients with FBS [3, 6].

Presentation of FBS usually occurs in early infancy but the late presentation has also been reported [7]. We found wide variability and severity even in patients within the same family or with the same pathogenic variant in *SLC2A2*, in keeping with other reports suggesting other factors that may underlie this that necessitate further studies [8, 9]. Six patients (60%) from ten unrelated families harbored the same p.(Arg53Ter) variant, suggesting this may be a founder variant in the Sudanese population and could be screened by genotyping before sequencing of the whole gene in Sudanese patients. Our patients presented late in the disease course with strong features of rickets, abdominal distention, and growth impairment. Many patients had a history of recurrent tachypnea and dehydration which were wrongly diagnosed as pneumonia or gastroenteritis at their first presentation to their local health facility, and six families reported that they had other children who died with a similar clinical picture without being diagnosed. Features of growth failure, hepatomegaly, signs of rickets, and tubulopathy are typical in FBS, but some other rare features have been described [10]. Though our patients had typical clinical features of FBS at presentation, abdominal distension remained the main concern that brought our patients to medical attention, while rickets or high blood glucose were incidentally discovered during the examination and investigations. One of our patients reported delayed dentition at the age of 16 months. This highlights the importance of careful dental assessment in patients with FBS where many abnormalities have been described and are explained by the presence of hypophosphatemic rickets and chronic acidosis [11]. Skeletal deformities in patients with FBS were not uncommon and severe chest deformity was seen in three of our patients. This may be explained by bone disease seen in patients with hypophosphatemic rickets as was proposed in similar reports [12].

The birth weight of patients with FBS has been found to be low in some reports, similar to our cases which may suggest low insulin in utero. This has not been widely discussed in the literature and further researches are needed to explain this observation [13]. Polyuria was documented as early as 40 days in eight patients of whom three proved to have neonatal diabetes. *SLC2A2* has been documented to be the cause of neonatal diabetes (NDM) in different reports suggesting the role of GLUT2 in insulin secretion [5]. Our patients with NDM were the outcome of consanguineous marriage, homozygous for the same *SLC2A2* mutation (c.157C > T) and had low birth weight suggesting reduced insulin secretion in utero, though normal birth weight has been reported before in patients with FBS and NDM [14]. Diabetes in this group tends to be transient suggesting low pancreatic B cell mass in the neonatal period that increases during development in infancy. Transient hyperglycemia is less severe suggesting that glycosuria frequently seen in FBS patients may help in decreasing the blood glucose level, however permanent NDM has also been reported [15]. It is not known why some patients of FBS who share the same mutations develop NDM while others do not, but it has been proposed that relatively late presentation and milder diabetes seen in FBS cases might be implicated as patients’ diabetes might have resolved by the time of the diagnosis [5].

Growth is greatly affected in patients with FBS and that was obvious in our patients at presentation. Delayed presentation and management with some patients having repeated episodes of chest infections and gastroenteritis could contribute to this. Other factors include proximal tubular dysfunction and glucose hemostasis disturbances. It has been proposed that nocturnal enteral nutrition (NEN) might improve the growth failure seen in patients with FBS [16]. We attempted corn starch feeding at night (at a dose of 1.6 g/kg) to our patients at diagnosis to prevent early morning hypoglycemia for which they showed good response [17].

Glycosuria, as seen in most of our tested patients, is moderate to severe at the time of diagnosis, however mild glycosuria in a milder phenotype of this syndrome has been reported [18]. The fasting blood glucose is found to be low in patients with FBS while they have a higher post prandial level. Interestingly, our patients showed a much lower values of fasting blood glucose and higher post prandial glucose levels compared to a previous study done to evaluate the blood glucose level in FBS patients. However, it may be that our patients were younger at presentation (0.9$\pm$0.8 years) than the other study (7.3 ± 4.8 years) and this could be explained by improving glucose levels over time [19]. Genetic diagnosis has decreased the need for a liver biopsy to confirm FBS diagnosis and such an invasive procedure can be avoided if genetic testing is available. Features on liver biopsy include deposition of glycogen in the hepatocytes, steatosis and fibrosis which were similar to our findings. Liver carcinoma is not known to be an association but a case of a liver carcinoma with FBS has been reported and the mechanism of this remains unknown [20].

Management is largely supportive and focused on the treatment of rickets and acid-base disturbance. In many developing countries medications such as phosphate, vitamin D analogs, or even solutions to correct acid-base balance are difficult to access and thus should be secured even at the local level. Some patients showed good response after treatment, with improvement of acidosis and features of rickets, but long term follow up is needed for further evaluation of their growth and puberty. Most of our patients came from remote areas with very limited access to tertiary facilities therefore follow up is usually interrupted. Survival to adulthood is favorable with normal fertility documented in one case report [21]. Early recognition and appropriate management may lead to a better prognosis while late diagnosis complicates the picture and increases the mortality usually because of respiratory infections or liver failure as seen in some reported cases including ours [22, 23].

## Conclusions

We have reported the first series of patients with FBS from Sudan, who have variable clinical presentation and genetic mutations including a novel one. This could have possibly resulted after diagnosing the first case and improving on professionals’ and parents’ awareness, yet many cases are likely being missed due to lack of access to tertiary health facilities and thus these should be secured. Increased awareness among pediatricians and easy access to research funded molecular genetic testing through help from international institutes have helped in the diagnosis of these cases. Further research is needed to explain the etiology of some of the manifestations such as growth failure and prognosis of diabetes. Sudan, a country with high consanguinity rate and variable ethnic groups is virgin soil for further genetic work. Research funded genetic testing is available for free for any family with suspected FBS at www.diabetesgenes.org.

## Data Availability

All data are available from the corresponding author on reasonable request.

## Abbreviations

FBS: Fanconi Bickel syndrome
GLUT 2: Glucose transporter 2
FBG: Fasting blood glucose
2 hr PPG: 2-hour post prandial blood glucose
NDM: Neonatal diabetes
NEN: Nocturnal enteral nutrition
